# *Polygonum cuspidatum* and Its Active Components Inhibit Replication of the Influenza Virus through Toll-Like Receptor 9-Induced Interferon Beta Expression

**DOI:** 10.1371/journal.pone.0117602

**Published:** 2015-02-06

**Authors:** Chao-jen Lin, Hui-Ju Lin, Ter-Hsin Chen, Yu-An Hsu, Chin-San Liu, Guang-Yuh Hwang, Lei Wan

**Affiliations:** 1 Department of Pediatrics, Changhua Christian Children's Hospital, Changhua, Taiwan; 2 Department of Life Science, Tunghai University, Taichung, Taiwan; 3 School of Medicine, Chung Shan Medical University, Taichung, Taiwan; 4 Department of Ophthalmology, China Medical University Hospital, Taichung, Taiwan; 5 School of Chinese Medicine, China Medical University, Taichung, Taiwan; 6 Graduate Institute of Veterinary Pathobiology, National Chung Hsing University, Taichung, Taiwan; 7 Institute of Molecular Medicine, National Tsing Hua University, Hsinchu, Taiwan; 8 Department of Neurology, Changhua Christian Hospital, Changhua, Taiwan; 9 Department of Biotechnology, Asia University, Taichung, Taiwan; 10 Department of Gynecology, China Medical University Hospital, Taichung, Taiwan; Temple University School of Medicine, UNITED STATES

## Abstract

Influenza virus infection is a global public health issue. The effectiveness of antiviral therapies for influenza has been limited by the emergence of drug-resistant viral strains. Therefore, there is an urgent need to identify novel antiviral therapies. Here we tested the effects of 300 traditional Chinese medicines on the replication of various influenza virus strains in a lung cell line, A549, using an influenza-specific luciferase reporter assay. Of the traditional medicines tested, *Polygonum cuspidatum* (PC) and its active components, resveratrol and emodin, were found to attenuate influenza viral replication in A549 cells. Furthermore, they preferentially inhibited the replication of influenza A virus, including clinical strains isolated in 2009 and 2011 in Taiwan and the laboratory strain A/WSN/33 (H1N1). In addition to inhibiting the expression of hemagglutinin and neuraminidase, PC, emodin, and resveratrol also increased the expression of interferon beta (IFN-β) through Toll-like receptor 9 (TLR9). Moreover, the anti-viral activity of IFN-β or resveratrol was reduced when the A549 cells were treated with neutralizing anti-IFN-β antibodies or a TLR9 inhibitor, suggesting that IFN-β likely acts synergistically with resveratrol to inhibit H1N1 replication. This potential antiviral mechanism, involving direct inhibition of virus replication and simultaneous activation of the host immune response, has not been previously described for a single antiviral molecule. In conclusion, our data support the use of PC, resveratrol or emodin for inhibiting influenza virus replication directly and via TLR-9–induced IFN-β production.

## Introduction

The influenza virus is one of the most infectious respiratory tract pathogens and is annually responsible for significant levels of morbidity, mortality, and economic loss [1]. In recent years, influenza infection has garnered considerable attention as a public health issue. This is largely a result of a novel swine-origin H1N1 influenza virus that emerged in Mexico in April 2009 and rapidly spread worldwide [2]. The H1N1 influenza outbreak was the first pandemic of the twenty-first century. In March 2013, the highly pathogenic novel reassortant avian-origin influenza A virus, H7N9, emerged in Shanghai and Anhui, China. This virus caused rapidly progressing lower respiratory tract infections [3].

Currently, there are two classes of antiviral drugs approved by the US Food and Drug Administration (FDA) to treat or prevent influenza virus infections: M2 ion channel inhibitors (amantadine and rimantadine) and neuraminidase inhibitors (oseltamivir and zanamivir) [4]. The use of M2 inhibitors is limited because of widespread drug resistance. Therefore, only neuraminidase inhibitors are widely used in the treatment of seasonal and pandemic influenza infections. However, oseltamivir-resistant viruses with the NA H275Y mutation were found to be widespread in seasonal H1N1 since 2007 and have circulated worldwide since the 2007–2008 influenza season [5,6]. The continuing threat of high spread efficiency and the potential emergence of more resistant strains necessitate the identification of new anti-influenza drugs to treat both seasonal and pandemic influenza.

One of the raw materials used for the production of oseltamivir is shikimic acid, which is derived from the traditional Chinese spice, star anise (*Illicium verum*) [7]. Medicinal plant extracts have been widely used in traditional medicine, and several plants have been investigated and shown to exhibit significant activity against the influenza virus [8,9], however, the molecular mechanisms of these herbs remain unclear and require further investigation.

*Polygonum cuspidatum* (PC), commonly called Japanese knotweed, is a member of the buckwheat family (Polygonaceae). Eight active compounds have been identified from PC extract: 2-methoxystypandrone (1); emodin (2); resveratrol (3); polydatin (4); emodin-8-O-beta-D-glucopyranoside (5); (E)-3,5, 12-trihydroxystilbene-3-O-beta-D-glucopyranoside-2'-(3",4",5"-trihydroxybenzoate) (6); and catechin-3-O-gallate (7); and rubiadin (8). PC has historically been used as a laxative, and occasionally as a food. Recent studies have reported that PC extract and its active components have antipyretic and analgesic activities [10–12].

Toll-like receptor 9 (TLR9) was first identified as the receptor for unmethylated cytosine-phosphate-guanine motifs in bacteria, and several TLR9 ligands have been identified in viruses and fungi [13,14]. TLR9 resides in endolysosomal compartments and is transduced via the adapter protein myeloid differentiation primary response gene (88) (MyD88), resulting in the activation of two different pathways.

The first pathway is a nuclear factor κB (NF-κB)-dependent pro-inflammatory cytokine pathway, and the second involves the activation of type I interferon (IFN) genes through IFN regulatory factor 7 (IRF7) phosphorylation [15,16]. IRF7 has been identified as a key regulator of IFN induction [17]. The type I IFN (alpha and beta IFN) response is one of the first lines of defense against viral infections. IFN-α/β were reported to exhibit antiviral activity when secreted by cells treated with heat-inactivated influenza A virus [18]. It has also been reported that influenza viruses are poor IFN-α/β inducers. This is because influenza viruses employ mechanisms to evade and antagonize the IFN-α/β response through the nonstructural protein 1 (NS1) gene [19].

In this study, we screened 300 antipyretic Chinese herbal medicines using a cell-based, high-throughput screening system. The active compounds identified during screening were also investigated for their mechanisms of action.

## Materials and Methods

### Cell culture and viruses

Madin-Darby canine kidney (MDCK) cells, human embryonal kidney (293T) cells and A549 lung cancer cells were obtained from the Bioresource Collection and Research Center (HsinChu, Taiwan) and cultured in Dulbecco’s modified Eagle medium (DMEM; Invitrogen, Carlsbad, CA, USA) containing 10% fetal bovine serum (Invitrogen) and incubated in a 5% CO_2_ humidified incubator. pREP4-FluA-Luc was transfected into 293T cells. Stable clones were selected with 250 μg/mL hygromycin B (Roche Diagnostics) and limiting dilution was performed to isolate clones with the highest luciferase activity when infected with influenza A virus. The influenza A/WSN/33 (H1N1) virus (WSN) was obtained from the American Type Culture Collection (Manassas, VA, USA).

### Influenza A reporter construction

The influenza A reporter (pREP4-FluA-Luc) was constructed according to the method of Lutz et al. [20]. Briefly, luciferase gene was amplified by primers: lucfor: 5′-ATACGTCTCGGGGAGTAGAAACAGGGTAGATAATCACTCACTGAGTGACATCGGTAAAATGGAAGACGCCAAAAACATAAAF-3′; lucrev: 5′-ATACGTCTCATATTAGTAGAAACAAGGGTATTTTTCTTTACAATTTGGACTTTCCGCCC-3′. The amplified fragment was then cloned into the pHH21 plasmid (a generous gift from Prof. Earl Brown, University of Ottawa, Ottawa, Ontario, Canada). The DNA fragment containing RNA polymerase promoter I, the luciferase reporter, and RNA polymerase I terminator was cloned into pREP4 (Invitrogen).

### Virus infection and assay procedure

A549 cells were seeded into 96-well plates (1×10^4^ cells/well) and incubated at 37°C and 5% CO_2_ for 24 h. The cells were then washed twice with phosphate buffered saline (PBS). Influenza A virus was diluted with PBS containing 0.2% bovine serum albumin to 10 multiplicity of infection (MOI)/100 μL. Cells were infected with 10 MOI H1N1 and centrifuged at 2000 rpm at room temperature for 1 h. Cells were washed twice with PBS and the test compounds were added. The 96-well plates were then incubated at 33°C and 5% CO_2_ for 24 h. The supernatants containing the replicated virus were then added to 293T cells stably transfected pREP4-FluA-Luc and incubated at 37°C and 5% CO_2_ for 24 h. Luciferase activity was determined by a luciferase assay system (Promega, Madison, WI, USA).

For the TLR9 inhibition assay, A549 cells were pre-treated with the TLR9 inhibitor, Super-iODN, (Enzo Life Sciences, Farmingdale, NY, USA) at 2 μM for 3 h before infection with the influenza virus. For IFN-β neutralization, anti-IFN-β antibodies (eBioscience, San Diego, CA, USA) were applied together with diluted influenza virus at a concentration of 10 μg/mL.

### Quantitation of IFN-β

Cell culture supernatants were harvested 24 h after treatment and used directly for measurement of IFN-β concentration. IFN-β concentrations were determined using human IFN-β ELISA kit (PBL Assay Science, Piscataway, NJ, USA).

### Western blotting

Western blotting was performed on A549 cells infected with H1N1 virus and treated with PC and its active components. Cells were harvested, washed with 10 mL of PBS three times, and lysed with mammalian protein extraction reagent (Thermo Fisher Scientific, Inc., Rockford, IL, USA) containing a phosphatase inhibitor and a protease inhibitor. Proteins were separated on a 12.5% sodium dodecyl sulfide-polyacrylamide gel electrophoresis (SDS-PAGE) gel and blotted onto a polyvinylidene difluoride membrane. The antibodies used to detect H1N1 hemagglutinin and neuraminidase were generated by immunization of Balb/C mice with H1N1 proteins (Fitzgerald Industries, Acton, MA, USA). For visualization, membranes were probed with an anti-mouse IgG secondary antibody conjugated to horseradish peroxidase. Binding was detected by a chemiluminescent substrate according to the manufacturer’s instructions (Thermo Fisher Scientific). Blots shown are representative of at least three individual experiments.

### Plaque reduction assay

MDCK cells were seeded into 6-well plates (1×10^6^ cells/well). After incubation at 37°C and 5% CO_2_ overnight, cells were washed twice with PBS and infected with H1N1 influenza virus (100 plaque forming units (PFU)/well) and kept on ice for 1 h. The cells were treated with PC or its active components in DMEM containing 0.3% agarose. The plate was incubated at 37°C and 5% CO2 for 72 h. The media were removed and the cells were fixed with 4% paraformaldehyde for 1 h at room temperature and subsequently stained with 0.1% crystal violet for 20 min at room temperature.

### Quantitative polymerase chain reaction (qPCR)

Total RNA was extracted by using an RNA isolation kit (Qiagen, Valencia, CA, USA), and 5 μg of RNA were reverse-transcribed using a reverse transcriptase for conversion to cDNA (Invitrogen). Primers and probes used for qPCR were selected from a predesigned probe system (Roche, Burgess Hill, UK). The RNA abundance was normalized to glyceraldehyde 3-phosphate dehydrogenase RNA in each sample.

## Results

**Fig 1:**
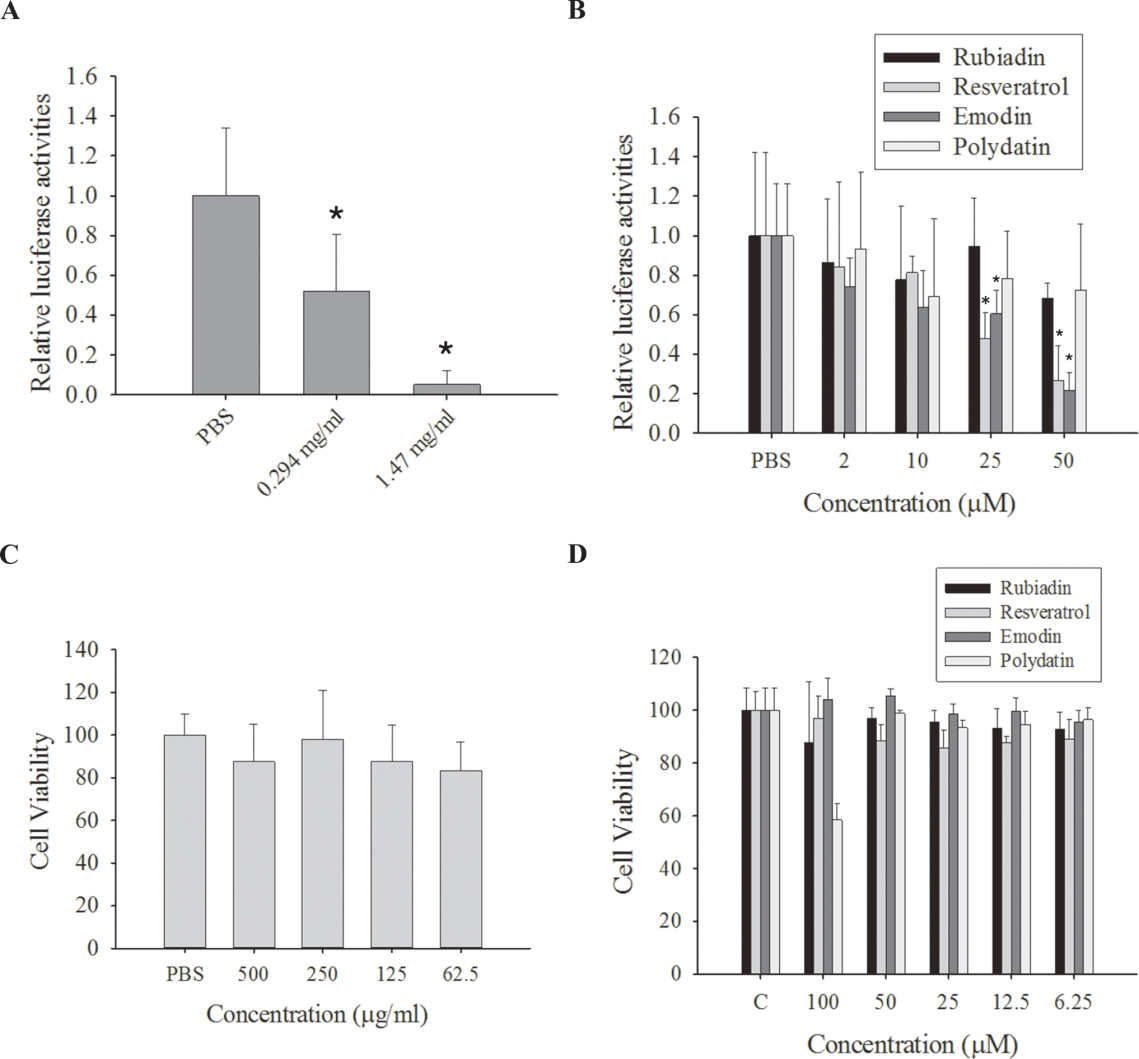
*Polygonum cuspidatum* and its active components inhibit replication of influenza A virus in A549 cells. (A) A water extract of *Polygonum cuspidatum* (PC) significantly inhibits influenza A virus replication in A549 cells. A549 cells were infected with 10 multiplicity of infection (MOI) H1N1 and treated with vehicle (phosphate buffered saline; PBS) or PC (0.294 and 1.47 mg/mL). The amplified virus in the culture supernatant was applied to 293T cells stably transfected with an influenza A reporter to determine influenza A replication in the presence of PC. (B) The active PC components rubiadin, resveratrol, emodin, and polydatin inhibited influenza A virus replication in A549 cells. A549 cells were infected with 10 MOI H1N1 treated with vehicle (PBS) or different concentration of rubiadin, resveratrol, emodin, and polydatin. The amplified virus in the culture supernatant was applied to 293T cells stably transfected with influenza A reporter to determine influenza A replication in the presence of rubiadin, resveratrol, emodin, and polydatin. Results are the mean ± standard deviation of three independent experiments performed in triplicate. Asterisks indicate the calculated p values for paired comparisons between control and drug treated samples are <0.05. (C and D) A549 cells were seeded into 96-well plates at a concentration of 5000 cells/well. Different concentrations of PC (C) or rubiadin, resveratrol, emodin, and polydatin (D) were added to each well. After 24 h, the cytotoxicity was determined by MTT assay. Results are the mean ± standard deviation of three independent experiments performed in triplicate.

The antiviral activities of PC were evaluated using a virus-inducible reporter system. H1N1 virus (10 MOI) was used to infect A549 lung cancer cells for 1 h, and the cells were then treated with a water extract of PC. The A549 culture supernatant containing the amplified H1N1 was then applied to 293T cells stably transfected with influenza A reporter plasmid (pREP4-FluA-Luc). PC inhibited H1N1 replication in A549 cells as shown by reduced luciferase activity (Fig. 1A). The IC_50_ value for PC was 312 μg/mL.

Four active ingredients of PC (rubiadin, resveratrol, emodin, and polydatin) were also tested to determine which compound(s) exhibited inhibitory effects on H1N1 replication in A549 cells. The IC_50_ values for rubiadin, resveratrol, emodin, and polydatin were >50 μM, 24.7 μM, 37.3 μM, and >50 μM, respectively. These results indicate that resveratrol and emodin were the most potent active compounds from PC for inhibition of H1N1 viral replication. We also determined the cytotoxicity of PC, rubiadin, resveratrol, emodin, and polydatin on A549 cells. PC, rubiadin, resveratrol, emodin, and polydatin exhibited low toxicity against A549 cells, with GI_50_ values of >500 μg/mL for PC (Fig. 1C) and >100 μM for rubiadin, resveratrol, emodin, and polydatin (Fig. 1D).

**Fig 2:**
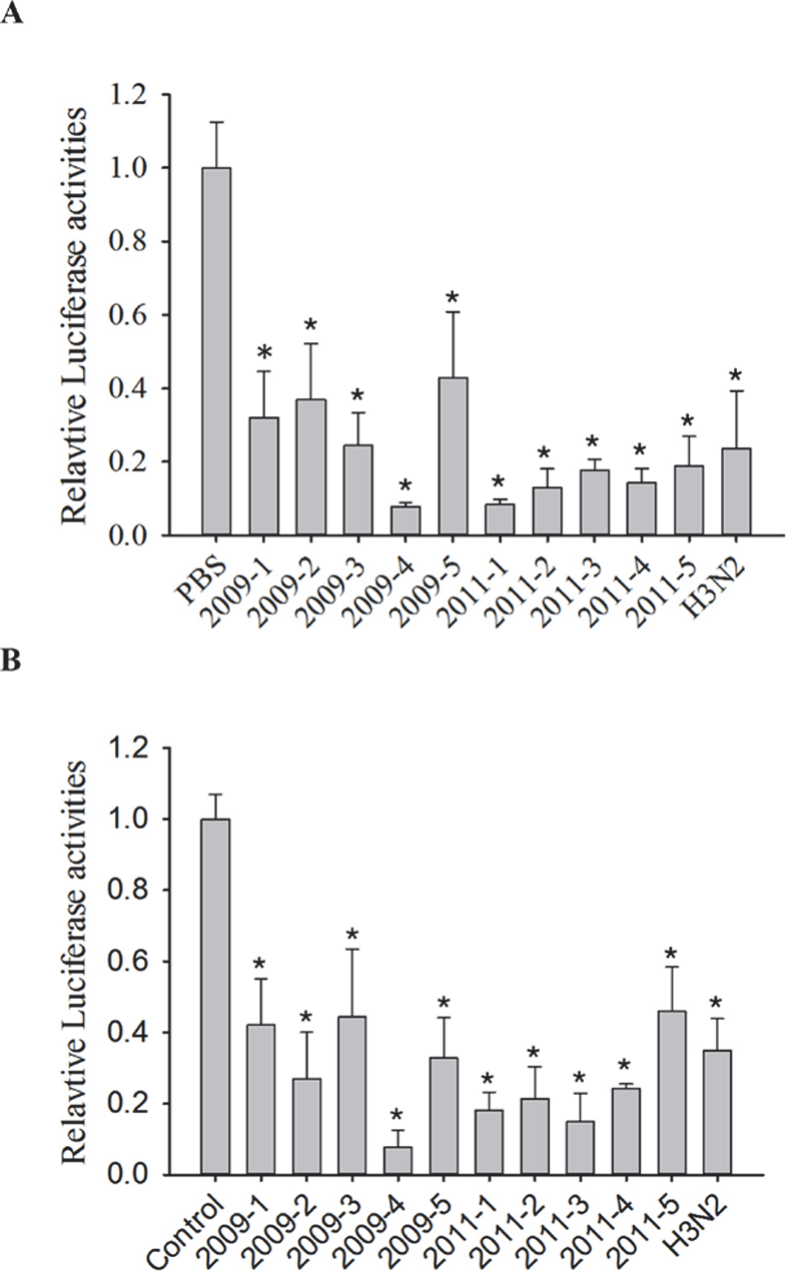
*Polygonum cuspidatum* (PC) and resveratrol inhibit the replication of H1N1 and H3N2 influenza A virus. (A) The water extract of PC significantly inhibited influenza A virus replication in A549 cells. A549 cells were infected with 10 multiplicity of infection (MOI) H1N1 treated with vehicle (phosphate buffered saline; PBS) or PC (0.3 mg/mL). The amplified virus in the culture supernatant was applied to 293T cells stably transfected with the influenza A reporter to determine influenza A replication in the presence of PC. (B) Resveratrol inhibits influenza A virus replication in A549 cells. A549 cells were infected with 10 MOI H1N1 treated with vehicle (PBS) or with 25 μM resveratrol. The amplified virus in the culture supernatant was applied to 293T cells stably transfected with influenza A reporter to determine influenza A replication in the presence of resveratrol. Results are the mean ± standard deviation of three independent experiments performed in triplicate. Asterisks indicate the calculated p values for paired comparisons between control and drug treated samples are <0.05.

One clinically isolated strain of H3N2 influenza virus (obtained in 2009) and 10 clinically isolated strains of H1N1 influenza viruses (collected in 2009 and 2011) were also treated with PC (Fig. 2A) and resveratrol (Fig. 2B). These compounds inhibited the replication of the clinical isolates with potencies similar to those observed for the laboratory strain of H1N1 influenza, A/WSN/33.

**Fig 3:**
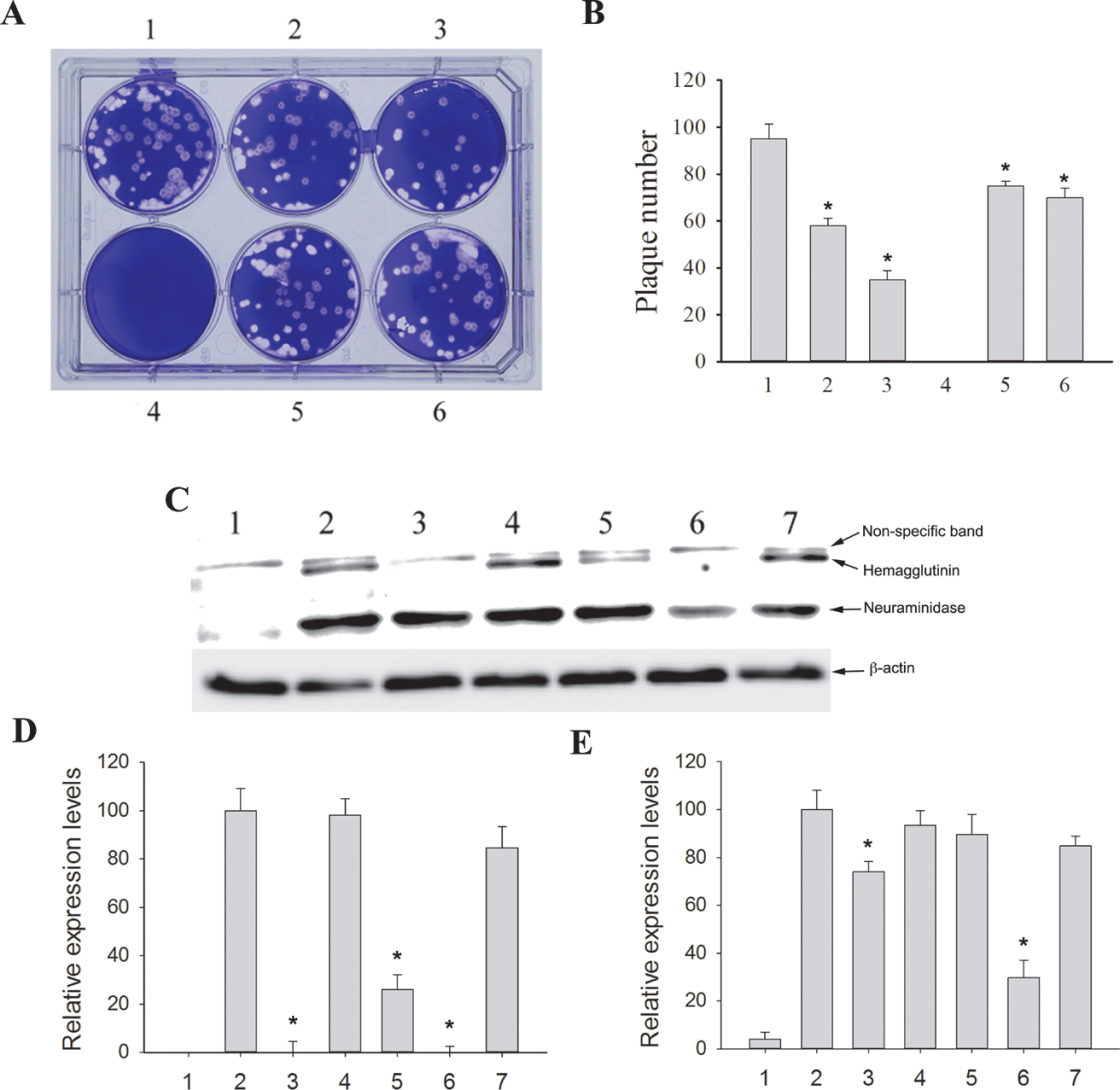
*Polygonum cuspidatum* (PC), resveratrol, and emodin reduced plaque numbers in a plaque reduction assay and inhibited the accumulation of hemagglutinin and neuraminidase in A549 cells. (A) *Polygonum cuspidatum* (PC), resveratrol, and emodin reduced plaque numbers in a plaque reduction assay. A representative plaque reduction assay is shown. Madin-Darby canine kidney (MDCK) cells were seeded into 6-well plates (1 × 10^6^ cells/well) and infected with H1N1 influenza virus (100 PFU/well). The cells were treated with PC or its active components in Dulbecco’s modified Eagle medium containing 0.3% agarose. After 72 h, the plaques were determined by staining with 0.1% crystal violet. Well 1: virus control; well 2: virus + PC (1.47 mg/mL); well 3: virus + 25 μM resveratrol; well 4: mock; well 5: virus + PC (0.294 mg/mL); well 6: virus + 25 μM emodin. (B) PC, resveratrol, and emodin reduced plaque numbers in a plaque reduction assay. Results are the mean ± standard deviation of three independent experiments performed in triplicate. Asterisks indicate the calculated p values for paired comparisons between 1 and 2, 3, 5, and 6 are <0.05. (C) PC and its active components inhibit the accumulation of hemagglutinin and neuraminidase in A549 cells. A549 cells were infected with H1N1 and treated with PC or its active components and the cell extracts were collected at 24 h post infection. Lane 1: mock; lane 2: virus only; lane 3: PC (1.47 mg/mL); lane 4: rubiadin (25 μM); lane 5: emodin (25 μM); lane 6: resveratrol (25 μM); lane 7: polydatin (25 μM). The result shown is one of the Western blots from three independent experiments. (D) Densitometry quantification of the hemagglutinin expression levels from the Western blot analyses. (E) Densitometry quantification of the neuraminidase expression levels from the Western blot analyses. Results are the mean ± standard deviation of three independent experiments performed in triplicate.

As shown in Fig. 3A and B, a plaque reduction assay was performed to confirm the antiviral activities of PC, resveratrol, and emodin. The plaque numbers were significantly reduced in response to 1.47 mg/mL PC (well 2), 0.294 mg/mL PC (well 5), 25 μM resveratrol (well 3), and 25 μM emodin (well 6). Emodin inhibited hemagglutinin expression (Fig. 3C; lane 5) while PC and resveratrol reduced hemagglutinin and neuraminidase expression (Fig. 3C; lanes 3 and 6; respectively). Treatment with PC and its active ingredients inhibited the expression of H1N1 hemagglutinin (Fig. 3D) and neuraminidase (Fig. 3E) in A549 cells infected with H1N1. Taken together, these data indicate that PC inhibited H1N1 replication in A549 cells.

**Fig 4:**
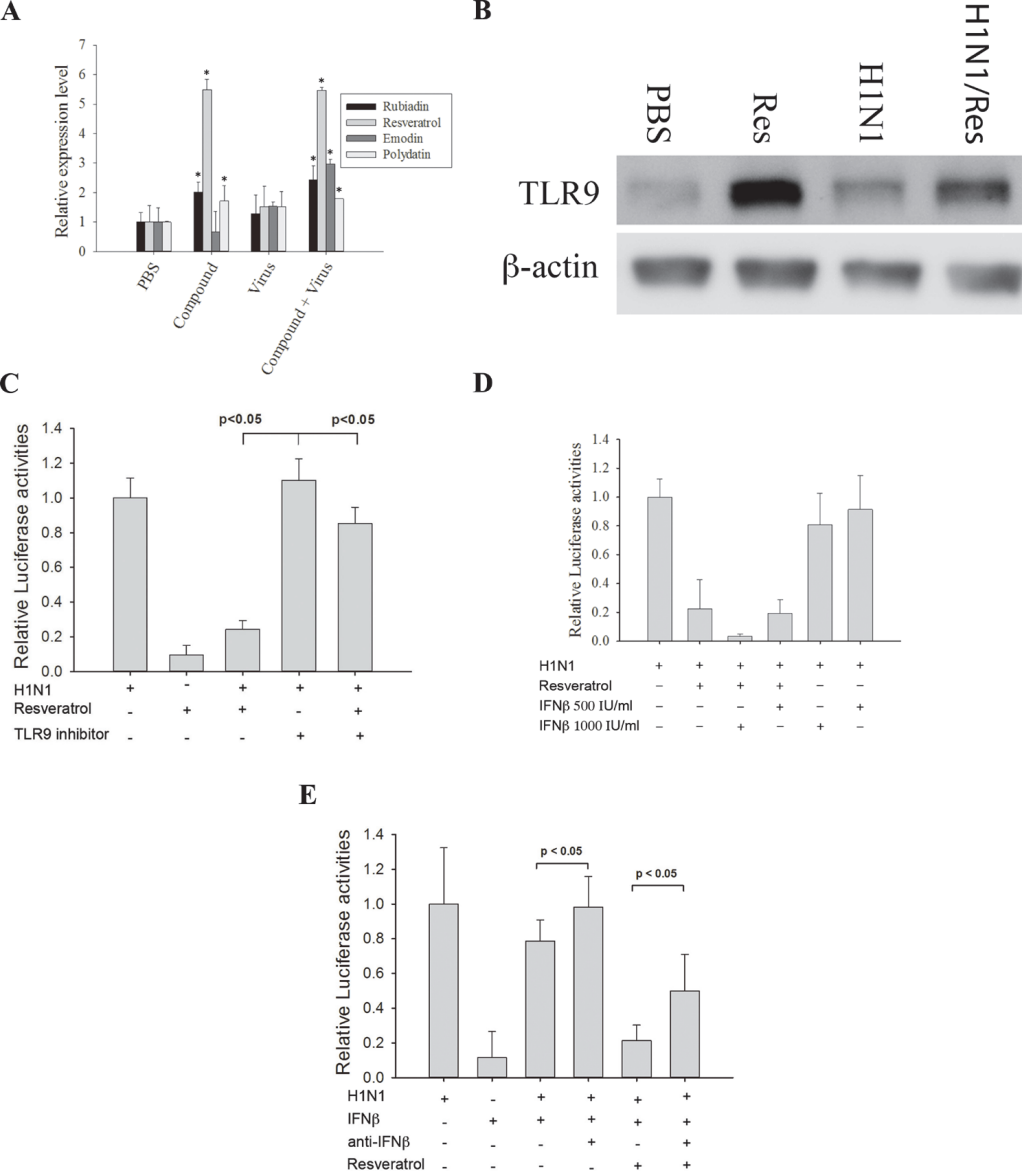
TLR9 and IFN-β acted synergistically with resveratrol to inhibit virus infection. (A) Resveratrol induces the expression of Toll-like receptor 9 (TLR9). A549 cells were treated as indicated and total RNAs were extracted to determine the TLR9 expression levels by real-time quantitative polymerase chain reaction (PCR). Results are the mean ± standard deviation of three independent experiments performed in triplicate. Asterisks indicate the calculated p values for paired comparisons between control (phosphate buffered saline; PBS) and drug treated samples are <0.05. (B) Resveratrol increased the expression level of TLR9. A549 cells were treated with PBS (control), 25 μM resveratrol, H1N1, H1N1 + 25 μM resveratrol and the cell extracts were collected at 24 h post infection. The result shown is one of the Western blots from three independent experiments. (C) Inhibiting TLR9 reduced the anti-H1N1 effect of resveratrol. A549 cells were treated with/without TLR9 inhibitor for 3 h before infected with H1N1. After 24 h of infection, the amplified virus in the culture supernatant was applied to 293T cells stably transfected with the influenza A reporter to determine influenza A replication in the presence of a TLR9 inhibitor. (D) Resveratrol works synergistically with interferon-β (IFN-β) to inhibit the replication of influenza A virus in lung cells. The treatments applied to A549 cells are listed in the figure. The amplified virus in the culture supernatant was applied to 293T cells stably transfected with influenza A reporter to determine influenza A replication in the presence of different treatments. The p values from Student’s t-tests for the paired comparisons of 25 μM resveratrol; 25 μM resveratrol + 1000 IU/mL IFN-β; 25 μM resveratrol + 500 IU/mL IFN-β versus the control are all <0.05. The p value from Student’s t-tests for the paired comparisons of 25 μM resveratrol versus 25 μM resveratrol + 1000 IU/mL IFN-β is <0.05. A p value < 0.05 is considered as significant. (E) Neutralizing IFN-β antibodies reduced the anti-viral activity of resveratrol. A549 cells were infected with 10 MOI H1N1. The treatments of A549 cells were listed in the figure. After 24 h of infection, the amplified virus in the culture supernatant was applied to 293T cells stably transfected with the influenza A reporter to determine influenza A replication in the presence of neutralizing anti-IFNβ antibodies. (F) A549 cells expressed IFN-β when treated with H1N1 or H1N1 + resveratrol. The treatments applied to the A549 cells are listed in the figure. After 24 h of infection, cell culture supernatants were collected to determine the IFN-β concentration (by ELISA). The p value from Student’s t-tests for the paired comparisons of H1N1 with H1N1 + resveratrol is <0.05. A p value <0.05 is considered as significant.

To investigate the underlying mechanisms of PC, emodin, and resveratrol in inhibiting influenza virus replication, we performed real-time PCR analysis for several antiviral-related genes. A549 cells were treated with PBS (control), the compounds alone, the virus alone, or the compounds and the virus. We found that TLR9 mRNA expression was increased when the cells were treated with PC, emodin, or resveratrol alone or when they were infected with virus and then treated with PC (294 μg/mL), emodin (25 μM), or resveratrol (25 μM). In contrast, the TLR9 mRNA expression level was unchanged when the cells were infected with the virus alone (Fig. 4A and Table 1). TLR9 protein expression level increased in A549 cells treated with resveratrol or resveratrol + H1N1, but was unchanged in cells infected with H1N1 alone (Fig. 4B).

**Table 1:** Gene expression levels of anti-virus replication related genes.

Gene Symbol	PBS	Virus	PC*	Virus/PC*	Emodin	Virus/Emodin	Resveratrol	Virus/Resveratrol
IFNA1	1±0.2	1.5±0.4	0.7±0.3	1.1±0.2	1.0±0.3	1.2±0.2	1.2±0.7	2.7±1.2
IFNB1	1±0.1	591.9±23.2^#^	0.3±0.1^#^ ^,^ ^&^	992.8±123.1^#^ ^,^ ^&^	0.3±0.1^#^ ^,^ ^&^	492.3±86.1^#^	0.8±0.1^#^ ^,^ ^&^	1093.7±98.1^#^ ^,^ ^&^
IRF3	1±0.5	1.1±0.4	1.6±0.4	3.2±1.2^#^ ^,^ ^&^	1.8±0.6	1.3±0.9	0.9±0.4	0.9±0.7
IRF5	1±0.2	0.8±0.3	2.5±0.9^#^ ^,^ ^&^	4.0±0.9^#^ ^,^ ^&^	2.1±0.6^#^ ^,^ ^&^	1.4±0.1^#^ ^,^ ^&^	2.3±0.2^#^ ^,^ ^&^	2.9±0.9^#^ ^,^ ^&^
IRF7	1±0.1	88.1±12.1^#^	4.0±1.1^#^ ^,^ ^&^	633.0±45.2^#^ ^,^ ^&^	4.5±1.6^#^ ^,^ ^&^	134.2±18.2^#^ ^,^ ^&^	3.9±0.8^#^ ^,^ ^&^	127.6±13.3^#^ ^,^ ^&^
ISG15	1±0.3	1126.3±90.1^#^	1.1±0.8^&^	2340.7±164.4^#^ ^,^ ^&^	1.8±0.8^&^	501.8±78.2^#^ ^,^ ^&^	2.1±0.9^&^	2462.7±154.2^#^ ^,^ ^&^
MX1	1±0.4	766.3±60.5^#^	1.8±0.6^&^	1026.1±79.1^#^ ^,^ ^&^	0.8±0.2^&^	258.7±34.1^#^ ^,^ ^&^	3.6±0.9^#^ ^,^ ^&^	1199.3±285.1^#^
MYD88	1±0.1	6.9±1.4^#^	1.8±0.4^#^ ^,^ ^&^	26.1±5.6^#^ ^,^ ^&^	0.7±0.4^&^	2.4±0.2^#^ ^,^ ^&^	0.9±0.5^&^	6.3±1.2^#^ ^,^ ^&^
NFKB1	1±0.2	2.8±0.98^#^	0.8±0.2^&^	1.5±0.3	0.6±0.3^&^	0.7±0.2^&^	1.1±0.9	2.4±3.2
NFKBIA	1±0.3	8.7±4.3^#^	2.4±0.6^#^	27.9±4.2^#^ ^,^ ^&^	7.1±3.6^#^	27.8±4.9^#^ ^,^ ^&^	1.7±1.1	6.8±1.4^#^
OAS2	1±0.2	2586. 0±123.1^#^	0.3±0.7^&^	2055.1±143.1^#^ ^,^ ^&^	0.3±0.1^#^ ^,^ ^&^	466.5±39.5^#^ ^,^ ^&^	2.0±1.8^&^	3422.9±453.1^#^ ^,^ ^&^
TLR3	1±0.4	42.6±14.3^#^	1.4±0.1^&^	46.7±9.8^#^	0.4±0.2^&^	2.9±0.5^#^ ^,^ ^&^	2.6±1.2^&^	69.6±24.2^#^
TLR7	1±0.1	13.7±4.2^#^	0.9±0.3^&^	3.7±1.6^&^	0.7±0.1^&^	1.1±0.5^&^	0.1±0.1^&^	3.2±1.6^&^
**TLR9**	**1±0.1**	**1.5±0.3**	**2.5±0.3** ^#^ ^,^ ^&^	**3.0±0.8** ^#^ ^,^ ^&^	0.7±0.7	**3.0±0.2** ^#^ ^,^ ^&^	**5.5±0.4** ^#^ ^,^ ^&^	**5.5±0.1** ^#^ ^,^ ^&^
TNF	1±0.2	45.8±12.1^#^	1.2±0.3^&^	47.8±8.1^#^	2.7±0.7^#^ ^,^ ^&^	53.8±10.1^#^	17.5±3.2^#^ ^,^ ^&^	138.6±54.1^#^ ^,^ ^&^
TRAF3	1±0.1	1.4±0.3	0.8±0.2^&^	1.2±0.4	0.5±0.1^#^ ^,^ ^&^	0.3±0.4^#^ ^,^ ^&^	1.3±0.4	1.6±0.8
TRAF6	1±0.3	1.2±0.7	0.9±0.7	1.0±0.4	0.5±0.2	0.4±0.8	1.2±0.2	1.6±0.7

*PC: *Polygonum cuspidatum*

＃indicate the calculated p values for paired comparisons between control and virus or drug or virus/drug treated samples are <0.05.

& indicate the calculated p values for paired comparisons between virus and drug or virus/drug treated samples are <0.05.

Results are the mean ± standard deviation of three independent experiments performed in triplicate.

The expression MYD88 mRNA was significantly increased with PC treatment. This is significant since MYD88 is involved in signal transduction resulting from TLR9 activation. It has been shown that TLR9 activates the IFN regulatory factors IRF3, IRF5, and IRF7 and the NF-κB family. IRF5 and IRF7 are strong transcriptional activators that lead to the expression of IFN-α and IFN-β. We found that PC, emodin, and resveratrol treatments significantly increased IRF5 and IRF7 mRNA expression (Table 1). The IRF7 mRNA levels were 7-fold higher when cells were treated with H1N1 + PC compared to A549 cells treated only with H1N1 (p < 0.05).

We also investigated the expression of IFNα and IFN-β. As shown in Table 1, IFN-β mRNA levels were two-fold higher in H1N1 + PC and H1N1 + resveratrol treated cells than in A549 cells only infected with H1N1 (p < 0.05). We did not observe a significant increase in IFN-α mRNA expression in this study. Together, these results indicate that IFN-β plays an important role in the clearance of H1N1 infection when treated with PC, emodin, and resveratrol.

Secreted type I IFN induces the expression of myxovirus-resistance protein 1 (MX1), which is important in inhibiting influenza A replication. MX1 mRNA expression levels increased 1.3-fold when cells were treated with H1N1 + PC or resveratrol compared to cells only infected with H1N1 (p < 0.05). In addition, interferon-induced protein IFI-15k (ISG15) and 2′,5′-oligoadenylate synthetase (OAS2) mRNAs exhibited expression patterns that were similar to MX1 (Table 1).

A specific TLR9 inhibitor, Super-iODN, was used to evaluate the importance of TLR9 in inhibiting influenza virus replication. We performed a reporter assay using resveratrol alone or in combination with the TLR9 inhibitor. A549 cells were infected with 10 MOI H1N1 virus for 1 h and subsequently treated with resveratrol, the TLR9 inhibitor, or resveratrol + the TLR9 inhibitor. Inhibition of H1N1 replication by resveratrol was reduced when the cells were treated with the TLR9 inhibitor (Fig. 4C). This indicated the importance of TLR9 in resveratrol mediated inhibition of replication.

To determine the effect of type I IFN on the inhibition of H1N1 replication, we performed a reporter assay using IFN-β alone or in combination with resveratrol. A549 cells were infected with 10 MOI H1N1 virus for 1 h and treated with resveratrol, IFN-β, or IFN-β + resveratrol. Our results indicate that IFN-β alone slightly inhibited viral replication in A549 cells (Fig. 4D). IFN-β combined with resveratrol produced more significant inhibition of H1N1 infection than resveratrol alone. The inhibition of H1N1 replication by IFN-β or IFN-β + resveratrol was reduced when cells were treated with neutralizing antibody to IFN-β (Fig. 4E).

The expression levels of IFN-β were also determined after A549 cells were treated with resveratrol. A549 cells were infected with 10 MOI H1N1 virus and treated with resveratrol, TLR9 inhibitor or TLR9 inhibitor + resveratrol. The expression level of IFN-β was significantly higher in cells treated with resveratrol compared with cells treated with TLR9 inhibitor or TLR9 inhibitor + resveratrol (Fig. 4F). These results indicate that resveratrol worked synergistically with IFN-β to inhibit H1N1 infection in A549 lung cells *in vitro*.

## Discussion

The aim of this study was to identify potential new treatments for influenza from traditional Chinese herbal medicines and their active components. We created an influenza A virus reporter cell system to screen potential candidates for inhibition of influenza virus replication. In addition, through investigation of the molecular mechanisms underlying these potential new treatments for influenza, we can assess potential new anti-virus pathways for developing more efficacious treatments.

In this study, we infected A549 cells with wild type influenza virus and treated them with various compounds. If a compound was effective in mitigating viral replication, viral RNA polymerase was present in lower amounts, leading to a concentration-dependent reduction in luciferase reporter levels. This indicated a concentration-dependent reduction in influenza virus replication.

Importantly, this screening process could be completed within 24 h instead of the 2–3 days necessary for plaque formation assays. These results indicated that our system was an effective method to assess influenza A viral titration. Notably, our reporter cell-based screening assay, as with similar assays, showed some level of toxicity to the cells by the test compounds, which could potentially lead to false positives. To avoid false positives, we performed MTT tests to exclude compounds that were toxic to the carrier cells.

In our small-scale screening program, we excluded toxic compounds and found that PC, emodin, and resveratrol exhibited significant inhibitory activity against H1N1 and H3N2 influenza A viruses with no significant toxic effect on the host cells. PC, resveratrol, and emodin exhibited potent antiviral activity against multiple subtypes of the influenza A virus, including the A/WSN/33 (H1N1) laboratory strain and the H3N2 and H1N1 clinical strains. Both of these clinically isolated strains have been shown to cause severe human respiratory disease. These strains were utilized in this study since both H3N2 and H1N1 have emerged during the twentieth century and caused four influenza A pandemics [21].

Hemagglutinin is responsible for the binding of the virus to the target cells to initiate viral infection, whereas neuraminidase cleaves the host cellular receptors and facilitates the release of the progeny virus, thus promoting the spread of the infection to neighboring cells [22,23]. Our western blots demonstrated that PC and two of its active components, emodin and resveratrol, reduced hemagglutinin and neuraminidase expression, indicating that they are capable of inhibiting viral replication.

The Type I IFN system (IFN-α/β) is the first line of defense against viral infections [24,25]. These molecules act directly on virus-infected cells, resulting in apoptosis, cytokine signaling, regulation of cell growth, and hematopoietic development [26]. PC, resveratrol, and emodin induced only IFN-β in the presence of influenza virus infection. Since IFN-β is likely to induce a specific antiviral immune response only in infected cells, this limits the likelihood that the undesirable side effects associated with systemic IFN therapy would be produced. Furthermore, since PC, resveratrol, and emodin induced IFN-β while retaining their direct antiviral activity, these synergistic effects may be a simple and efficient way to reduce influenza viral yield. This also suggests that the antiviral mechanisms of the compounds may be dependent on type I IFN. Therefore, these data indicate that the activation of an innate immune response was secondary to a more direct antiviral function of these compounds.

The viral nonstructural protein 1 (NS1) of the influenza A virus prevents the induction of the IFN-β promoter by inhibiting the activation of transcription factors such as IRF3 [27]. Both IRF3 translocation and NF-κB activation are impaired in the presence of NS1, which blocks the induction of proinflammatory cytokines and IFNs [28,29]. Our results show that IRF3 and NF-κB were not induced, indicating that the antiviral mechanisms of PC, resveratrol, and emodin may not involve the NS1 protein.

TLR9 is involved in cellular antiviral mechanisms. TLR9 activates two distinct pathways: the NF-κB-dependent proinflammatory cytokine pathway and the IRF7-dependent type I IFN pathway [19,30,31]. IRF7 directly interacts with the MYD88 signaling adaptor, and engagement of TLR9 by non-replicating viral genomic content leads to the rapid secretion of IFNs. Within the IRF family, IRF3 and IRF7 have been identified as key regulators of the IFN induction. However, IRF7 is largely responsible for IFN production in response to influenza A infection, as shown by the abrogation of IFN production in IRF7^-/-^ mice [32]. Our results indicate that the suppression of influenza virus replication and enhancement of IFN-β gene expression induced by PC, resveratrol, and emodin might be mediated through the regulation of IRF7.

**Fig 5:**
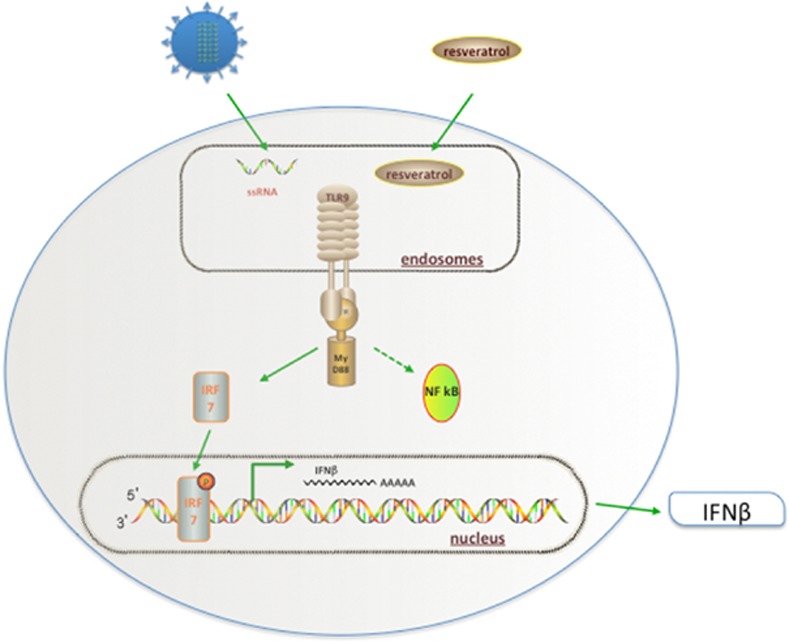
The molecular mechanisms underlying the antiviral activity of *Polygonum cuspidatum* (PC) and its active components. PC, resveratrol, and emodin enter the influenza-infected cell and then activate proinflammatory cytokines through TLR9. TLR9 signals through the adapter protein myeloid differentiation primary response gene 88 (MYD88), leading to the activation of two different pathways. PC, resveratrol, and emodin likely activate interferon regulator factor 7 (IRF7) pathway rather than the nuclear factor κB (NF-κB) pathway, leading to the activation of type I IFN genes through the phosphorylation of IRF7 and the transcriptional activation of proinflammatory cytokines.

In this study, we detected significantly elevated levels of TLR9 and reduced influenza virus A replication in cells treated with PC, resveratrol, or emodin compared to control cells (P < 0.05). This suggests that enhanced TLR9 production and the activation of virus-induced IRF7 induced IFN-β protein expression. Taken together, these data demonstrate that PC, resveratrol, or emodin, in the presence of the influenza A virus, result in the activation of IFN-β through the TLR9-MYD88–IRF7 pathway (Fig. 5).

In this study, we identified anti-influenza A virus compounds by screening 300 Chinese herbal medicines in a luciferase reporter assay and found that the Chinese herbal compound, PC, and two of its active components, resveratrol and emodin, induced IFN-β only in the presence of influenza virus infection. Moreover, these three compounds exhibited a superior ability to induce the expression of IFN-β and ISGs. PC, resveratrol, and emodin inhibited the growth of influenza A viruses, including the clinically isolated strains H3N2 and H1N1 and the A/WSN/33 (H1N1) laboratory strain. We found that the antiviral activities of PC, resveratrol, and emodin were dependent on IFN production and signaling. This inhibitory effect was associated with the induction of IFN gene expression through the TLR9-MYD88–IRF7 pathway. Together, the findings of this study suggest that synergistic effects of antiviral compounds and IFN-β can protect against influenza virus infections. These insights may lead to the development of novel antiviral therapies for the treatment of influenza.
