# Correction: *Polygonum cuspidatum* and Its Active Components Inhibit Replication of the Influenza Virus through Toll-Like Receptor 9-Induced Interferon Beta Expression

**DOI:** 10.1371/journal.pone.0125288

**Published:** 2015-04-10

**Authors:** Chao-jen Lin, Hui-Ju Lin, Ter-Hsin Chen, Yu-An Hsu, Chin-San Liu, Guang-Yuh Hwang, Lei Wan

Fig 4, “TLR9 and IFN-β acted synergistically with resveratrol to inhibit virus infection” is incorrect. Please see the corrected Fig 4 and it’s legend here:

**Fig 4:**
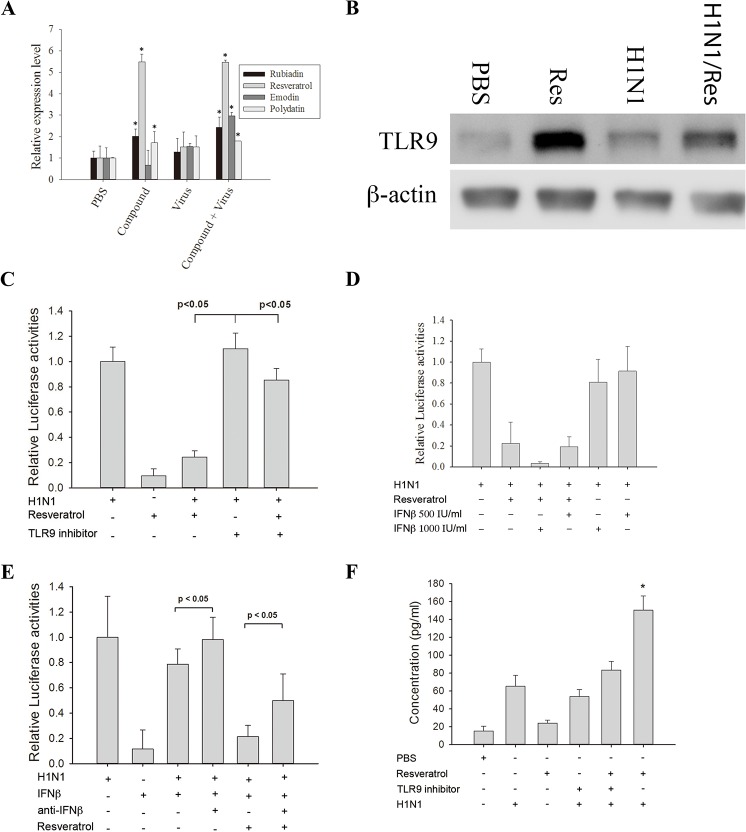
TLR9 and IFN-β acted synergistically with resveratrol to inhibit virus infection. (A) Resveratrol induces the expression of Toll-like receptor 9 (TLR9). A549 cells were treated as indicated and total RNAs were extracted to determine the TLR9 expression levels by real-time quantitative polymerase chain reaction (PCR). Results are the mean ± standard deviation of three independent experiments performed in triplicate. Asterisks indicate the calculated p values for paired comparisons between control (phosphate buffered saline; PBS) and drug treated samples are <0.05. (B) Resveratrol increased the expression level of TLR9. A549 cells were treated with PBS (control), 25 μM resveratrol, H1N1, H1N1 + 25 μM resveratrol and the cell extracts were collected at 24 h post infection. The result shown is one of the Western blots from three independent experiments. (C) Inhibiting TLR9 reduced the anti-H1N1 effect of resveratrol. A549 cells were treated with/without TLR9 inhibitor for 3 h before infected with H1N1. After 24 h of infection, the amplified virus in the culture supernatant was applied to 293T cells stably transfected with the influenza A reporter to determine influenza A replication in the presence of a TLR9 inhibitor. (D) Resveratrol works synergistically with interferon-β (IFN-β) to inhibit the replication of influenza A virus in lung cells. The treatments applied to A549 cells are listed in the figure. The amplified virus in the culture supernatant was applied to 293T cells stably transfected with influenza A reporter to determine influenza A replication in the presence of different treatments. The p values from Student’s t-tests for the paired comparisons of 25 μM resveratrol; 25 μM resveratrol + 1000 IU/mL IFN-β; 25 μM resveratrol + 500 IU/mL IFN-β versus the control are all <0.05. A p value < 0.05 is considered as significant. (E) Neutralizing IFN-β antibodies reduced the anti-viral activity of resveratrol. A549 cells were infected with 10 MOI H1N1. The treatments of A549 cells were listed in the figure. After 24 h of infection, the amplified virus in the culture supernatant was applied to 293T cells stably transfected with the influenza A reporter to determine influenza A replication in the presence of neutralizing anti-IFNβ antibodies. (F) A549 cells expressed IFN-β when treated with H1N1 or H1N1 + resveratrol. The treatments applied to the A549 cells are listed in the figure. After 24 h of infection, cell culture supernatants were collected to determine the IFN-β concentration (by ELISA). The p value from Student’s t-tests for the paired comparisons of H1N1 with H1N1 + resveratrol is <0.05. A p value <0.05 is considered as significant.
